# Protective role of dietary zinc on DNA damage, oxidative stress, and metal toxicity

**DOI:** 10.3389/fmolb.2025.1618318

**Published:** 2025-06-24

**Authors:** Carmen P. Wong, Laura M. Beaver, Emily Ho

**Affiliations:** ^1^ Linus Pauling Institute, Oregon State University, Corvallis, OR, United States; ^2^ School of Nutrition and Public Health, Oregon State University, Corvallis, OR, United States

**Keywords:** zinc, zinc deficiency, DNA damage, oxidative stress, heavy metals toxicity

## Abstract

Dr. Bruce Ames illustrious career spanned many decades with far-reaching impacts both on our knowledge of DNA and in public health. In the 1990s he explored the connection between inadequate intake of micronutrients and single- and double-strand DNA breaks, genome instability due to oxidative damage, and increased susceptibility to cancer and other age-related diseases. In particular, zinc is an essential micronutrient required for many biological processes and is a key component of numerous proteins and enzymes involved in the defense against oxidative stress and DNA damage repair. Reduced zinc status due to inadequate dietary intake, reduced zinc absorption and increased excretion can lead to increased risks for infectious diseases, diabetes, cancer, and neurological disorders. Changes in zinc status can also positively or negatively modulate the outcome of exposure to toxic heavy metals including arsenic, cadmium, and lead. This mini review highlights the role of zinc in maintaining DNA integrity and antioxidant defense, the health consequences of inadequate zinc intake, and the impact of zinc status on the response to environmental exposure to toxic metals. Collectively, the work by Dr. Ames and others advances our understanding of how zinc status plays an integral role in health, and reaffirms the idea originally put forth by Dr. Ames that optimizing micronutrient intake to ensure adequate nutrition, including zinc intake, is essential in promoting health, longevity, and disease prevention.

## Introduction

Dr. Bruce Ames illustrious scientific contributions ranged from understanding the relationship between mutagenesis and carcinogenesis, to the role of oxidative stress and DNA damage plays in mitochondrial decay and aging (Ames, 2022). Early in his career, his research focused on the study of the genetic, enzymological, and regulatory aspects of the large and complicated histidine biosynthetic pathway in *Salmonella typhimurium* in the 1960s and ultimately led to the development of the Ames Test for mutagens. This test is widely used as a sensitive and cost-effective tool for identifying compounds with mutagenic potential from both synthetic and natural sources. In the 1990s he became interested in the connection between DNA damage and cancer, inflammation, and oxidative damage, which led to further ideas about poor quality diets and micronutrient deficiency being a major contributor to DNA damage and cancer.

Work by Dr. Ames and colleagues in the early 2000s indicated inadequate intake of micronutrients (including vitamins B12, B6, C, E, folate, niacin, iron and zinc) can cause single- and double-strand DNA breaks, genome instability due to oxidative damage, and accelerate mitochondrial oxidative decay associated with aging (Ames, 1999; 2001; Ames et al., 2005). Dr. Ames published the Triage Theory in 2006 that postulates “DNA damage and late onset disease are consequences of a triage allocation response to micronutrient scarcity” and when micronutrients are limiting, processes that favor short-term and immediate survival are favored at the expense of systems that maintain long term health like antioxidant function and DNA repair mechanisms (Ames, 2006). The Triage Theory provides a causal link by which inadequate micronutrient intakes lead to DNA damage, loss of mitochondrial function, accelerated aging and age-associated chronic diseases. Moreover, ensuring adequate micronutrient intake through diet and supplementation may mitigate the triage process. Micronutrient inadequacies, defined as nutrient intake less than the Estimated Average Requirement (EAR), is pervasive worldwide and is a global public health concern (Passarelli et al., 2024). Dr. Ames’ research brought attention to the importance of consuming optimal nutrients for achieving health, longevity, and disease prevention. He was a strong proponent in remedying micronutrient inadequacies via optimizing micronutrient intake (through diet and dietary supplements) to reduce the risks of age-related chronic diseases and promote healthy aging.

Among the various nutrients Dr. Ames studied, zinc is an essential micronutrient required for many biological functions including growth and development, cognitive function, reproduction, bone health, and immunity (Maywald and Rink, 2022). Zinc homeostasis is tightly regulated by zinc transporter family members, with zinc metabolism and signaling playing critical roles in many cellular processes (Hara et al., 2017; Chen et al., 2024). In particular, zinc is a key component of numerous proteins and enzymes involved in the defense against oxidative stress and DNA damage repair. In humans, decrease in zinc status can result from low dietary intake of zinc, inadequate zinc absorption, increased zinc excretion, or an increased need for zinc. While severe zinc deficiency caused by low dietary zinc intake is uncommon in high income countries, mild zinc deficiency is potentially prevalent worldwide (Lowe et al., 2024). In the United States, it is estimated that 15% of US adults have zinc intakes below the EAR, with select groups of individuals particularly at risk for zinc deficiency (e.g., children, pregnant or lactating women, adults ≥65 years of age, and individuals with certain chronic diseases) (Reider et al., 2020; NIH-ODS, 2022). Alteration in zinc status biomarkers (serum zinc concentrations) or zinc intake (dietary zinc intake or zinc supplementation) are associated with a variety of health outcomes (Li J. et al., 2022). For example, reduced zinc status is associated with increased risks for infectious diseases (Maywald and Rink, 2022), diabetes (Tamura, 2021), cancer (Sugimoto et al., 2024), and neurological disorders (Li Z. et al., 2022). At the same time, increased dietary zinc intake is associated with decreased risk for certain cancers, depression, and diabetes; while zinc supplementation improved depression symptoms, increased pregnancy rate, and decreased concentration of inflammatory markers (Li J. et al., 2022).

The work from Dr. Ames advanced our understanding of how maintaining adequate zinc status plays an integral role in health. In this mini review, we highlight and update the work by Dr Ames and colleagues since the proposal of the Triage Theory, with focus on the role of zinc in maintaining cellular antioxidant defense and DNA integrity. In the presence of low cellular zinc, due to the Triage Theory, the ability to adequately respond to stressors is impaired due to loss of antioxidant and DNA repair functions. We connect the impact of zinc status on environmental toxic metals exposure, as this highlights the interaction between micronutrient status and environmental factors that together influence health outcomes.

## The Role of Zinc in Maintaining DNA Integrity and Antioxidant Defense

As proposed by Dr. Ames, the triage response to inadequate micronutrient intakes, including zinc, results in impaired antioxidant defense and oxidative DNA damage. Work by Dr. Ames and others has demonstrated the role of zinc in protecting cellular components from oxidative damage in a variety of cell lines and animal studies. Zinc has a well-established role in antioxidant defense and mediates its protective role against oxidative damage via multiple mechanisms. Zinc 1) serves as a cofactor for enzymes involved in the functioning of the antioxidant defense system; 2) affects cellular redox balance by inducing the synthesis of metallothionein and glutathione; 3) protects against protein sulfhydryl groups oxidation; and 4) inhibits the pro-oxidant enzyme NADPH-oxidase that generates reactive oxygen species (ROS) (Lee, 2018). Zinc also plays an important role in maintaining DNA integrity. It is required in the regulation of DNA replication via zinc finger proteins, affects chromatin accessibility and transcription factor binding to DNA, and is involved in DNA damage response and repair (Yan et al., 2008; Ocampo et al., 2024). Low intracellular zinc induces DNA damage in cells via a combination of increased oxidative DNA damage and disruption of zinc-dependent proteins involved in DNA-repair pathways, leading to impaired DNA repair and altered expression of DNA damage response genes, resulting in DNA strand breaks and genome instability (Ho and Ames, 2002; Ho et al., 2003; Yan et al., 2008; Sharif et al., 2012). In particular, zinc-dependent transcription factors such as p53, a critical gatekeeping factor in coordinating the response to DNA damage, was impacted with cellular zinc deficiency where loss of zinc in the DNA binding domain impaired DNA binding capacity and compromised function (Ho and Ames, 2002; Yan et al., 2008). Observations from cell culture models were supported by animal models, where severe zinc deficiency induced by dietary zinc restriction similarly caused oxidative stress, DNA damage, and impaired DNA repair and antioxidant defense responses (Oteiza et al., 1995; Bruno et al., 2007; Song et al., 2009b). In more recent animal studies zinc deficiency exacerbated age-related DNA damage by impairing the catalytic activity of 8-oxoguanine DNA glycosylase, an enzyme involved in the base excision repair pathway (Sharma et al., 2024). In other recent studies, zinc deficiency-mediated oxidative stress led to increased inflammation and fibrosis in the lung, induced inflammation and apoptosis in the kidney in zinc deficient animals, and exacerbated age-related chronic inflammation in old mice (Wong et al., 2021; Zhang et al., 2022; Xu et al., 2023).

To model inadequate zinc intake prevalent in human populations, both severe and marginal zinc deficient diets have been used in animal studies to examine various health outcomes, including the effects on DNA damage and integrity. While severe zinc deficiency caused more damage, animals with marginal zinc deficiency similarly had increased oxidative stress, impaired DNA integrity and DNA repair functions, and increased DNA damage compared to animals in the zinc adequate group (Song et al., 2009b). Marginal zinc deficiency also sensitized animals to increased oxidative DNA damage after chronic exercise (Song et al., 2010a), altered zinc transporter expression and zinc homeostasis in the prostate (Song et al., 2010b), increased age-related chronic inflammation (Wong et al., 2021) and enhanced toxicity associated with exposure to heavy metals (see next section). Importantly, zinc repletion studies showed the deleterious effects of zinc deficiency on DNA integrity can be reversed via dietary intervention. The effects of dietary zinc depletion and repletion in rats showed the increase in DNA damage induced with low zinc intake can be normalized with zinc repletion that restored DNA integrity (Song et al., 2009b). Notably, similar observations were reported in human studies. In one human study, increased DNA strand breaks in peripheral blood cells associated with dietary zinc depletion were ameliorated by zinc repletion (Song et al., 2009a). In other human studies, a moderate increase in dietary zinc (Zyba et al., 2017) or daily oral zinc supplementation (Joray et al., 2015) resulted in a reduction in DNA strand breaks in leukocytes of individuals with improved zinc status. Collectively, these studies reaffirm Dr. Ames’ Triage Theory, and showed dietary zinc deficiencies contribute to oxidative stress and DNA damage that can be reversed via dietary interventions. This underscores the role of zinc in maintaining DNA integrity and health, and the potential for disease prevention via increased zinc intake.

## Zinc status and toxic metals exposure in the environment

Zinc deficiencies often occur in human populations in regions of the world that are also co-exposed to toxic heavy metal contaminants (Wong et al., 2019). In addition to direct health consequences (Triage response) attributed to inadequate micronutrient intake, an extension to the Triage Theory is that micronutrient deficiencies such as inadequate zinc can impair the cellular responses to environmental stresses, such as toxic metals exposure to exacerbate negative health outcomes. In this section, we discuss how zinc deficiency and zinc supplementation alter response to toxic metals exposure in cell culture, animals, and human studies.

Chronic environmental and occupational exposure to some heavy metals and metalloids cause diverse toxic effects in the body (Balali-Mood et al., 2021). According to the World Health Organization, lead, cadmium, mercury and arsenic are among the top 10 chemicals of major health concern (WHO, 2020). Data from 2007 to 2012 NHANES survey indicated that approximately 50% of the US population was exposed to a combination of three or more of these toxic metals (Shim et al., 2017). This has significant impact on public health as exposure to cadmium, lead, and arsenic can cause liver damage, injury to the central nervous system and lungs, and has been associated with gastrointestinal disorders, immune dysfunction, kidney dysfunction, cardiovascular dysfunction, degenerative bone disease, birth defects, cancer, and an increase in all-cause mortality (Balali-Mood et al., 2021; Guo et al., 2022). The toxic effects of exposure to heavy metals are complicated and dependent on the exposure route, form of the metal, and the dose and duration of exposure (Tchounwou et al., 2012). It is also notable that the effects of toxic metal exposure are dependent on the characteristics of the person exposed, with factors like age, gender, and nutritional status (e.g., zinc status) as determining factors if toxicity is observed (Tchounwou et al., 2012). Further, tissue accumulation of heavy metals and associated organ-specific toxicity can be modulated by the expression and activity of metal transporters, including zinc transporters (Dashner-Titus et al., 2023; Ferdigg et al., 2025).

**FIGURE 1:**
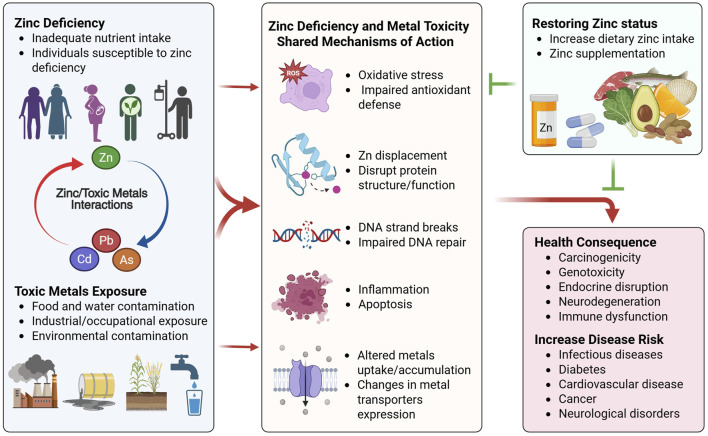
Interactions between zinc status and toxic metals exposure. Populations at risk for zinc deficiency and toxic metals exposure are susceptible to cellular and organ damage via common mechanisms. Zinc deficiency and toxic metals exposure both lead to increased oxidative stress and impaired antioxidant defense, resulting in impaired DNA repair, DNA strands breaks, inflammation and apoptosis. This is in part mediated via displacement of zinc and disruption of zinc finger protein structure and function, and dysregulated expression of metal transporters that alter zinc homeostasis and toxic metal uptake and accumulation. These common interactions between zinc deficiency and toxic metals exposure can exacerbate metal-induced toxicity when both conditions are present, resulting in further cellular damage, increased genotoxicity and carcinogenicity, disruption of cellular and organ functions, and increased risk for various chronic diseases. Improving zinc status via increased dietary zinc intake and/or zinc supplementation can ameliorate cellular damage and confer protection against toxic metals exposure. Created in BioRender. Wong, C. (2025) https://BioRender.com/mdjt9mx.

The mechanisms by which metals cause damage and genotoxicity at the cellular level include: 1) Increasing ROS production and decreasing antioxidant defense, causing DNA, protein, and lipid damage; 2) displacing zinc from zinc finger proteins and disrupting DNA repair, cell division, and other zinc-dependent processes; 3) induction of endoplasmic reticulum stress and mitochondrial dysfunction; and 4) induction of inflammation and apoptosis (Banerjee et al., 2020; Balali-Mood et al., 2021; Koyama et al., 2024). Notably many of the cellular processes negatively affected by toxic metal exposure overlap significantly with zinc deficiency (Figure 1). This suggests potential interaction between zinc and toxic metal exposure, whereby changes in zinc status can positively or negatively modulate the outcome of metal toxicity (Hudson et al., 2025). In general, increased toxicity is observed from toxic metal exposure under the conditions of zinc deficiency, while zinc supplementation has been shown in some models to protect from heavy metal exposure (Wani et al., 2021; Hudson et al., 2025). The toxic metals that have been studied the most in the context of nutritional zinc status are cadmium, arsenic, and lead and are the focus of this review.

## Zinc deficiency increases toxicity from cadmium, arsenic and lead exposures

The strongest evidence that zinc deficiency exacerbates metal-induced toxicities are from *in vitro* and animal studies. For example, in cell culture models zinc deficiency and arsenic co-exposure increased levels of ROS, DNA strands break, apoptosis, and inflammation, beyond what was observed with either condition alone (Cao et al., 2019; Wong et al., 2019). Further, zinc deficiency exacerbated lead-induced suppression of interleukin-2 production in immune cells (Trojan et al., 2024). Cellular zinc loss also enhanced the cytotoxicity of lead in neuronal cells by further reducing cell viability and increased the intracellular oxidant levels that led to activation of oxidant-responsive transcription factors, such as AP-1 that contributed to neuronal cell death (Aimo and Oteiza, 2006). In rats, dietary zinc deficiency increased lead and cadmium accumulations in various organs, resulting in impaired skeletal growth, increased neoplastic progression of testicular lesions and enhanced chronic progressive nephropathy (Bushnell and Levin, 1983; Waalkes, 1986; Waalkes et al., 1991; Jamieson et al., 2006). In mice, co-exposure of arsenic and zinc deficiency resulted in increased oxidative stress, DNA damage, and inflammation (Gaulke et al., 2018; Wong et al., 2019). Further, arsenic-induced perturbations in the gut microbiome was amplified with zinc deficiency, likely increasing the microbiome’s sensitivity to arsenic exposure and by altering the response of the microbiome to chemical exposure (Gaulke et al., 2018). In zebrafish, arsenic exposure significantly reduced the amount of zinc in the developing embryo, and zinc deficiency and arsenic co-exposure caused changes in the expression of genes that regulate zinc homeostasis, response to oxidative stress and insulin production, and decreased larval photomotor response, an assay used to assess neurotoxicological behavioral responses. The decline in larval behavior was significantly greater than what was observed with zinc deficiency or arsenic exposure alone (Beaver et al., 2017). Together these data suggest that zinc deficiency may sensitize cells and individuals to various toxic metal exposures.

## Zinc supplementation protects against cadmium, arsenic and lead exposures

*In vivo* zinc supplementation studies demonstrated increasing zinc status protected against various metals toxicities (Wani et al., 2021; Yu et al., 2021; Banerjee et al., 2022; Hudson et al., 2025). One mechanism by which zinc mitigates metal toxicity is by reducing toxic metal accumulation. In mice chronically exposed to arsenic, zinc supplementation reduced the amount of arsenic detected in all tissues tested, in part by modulating the expression of metal transporters (Dashner-Titus et al., 2023). Similar reductions in heavy metal accumulation and toxicity was observed in animals exposed to cadmium (Pabis et al., 2018) and lead (Hietanen et al., 1982; Ugwuja et al., 2020; Butt et al., 2023). Reduced toxic metal tissue burden can be mediated by zinc’s competition for metal cellular uptake, and its effect on the expression of metal transporters that affect metal absorption, accumulation, and excretion (Dashner-Titus et al., 2023; Ozoani et al., 2024). Another mechanism by which zinc reduces metal toxicity is via the induction of metallothionein expression and reduction of oxidative damage by upregulating and/or restoring antioxidant pathways. Metallothioneins are small proteins that function as antioxidants, scavengers of ROS, sequester toxic metals, and restore antioxidant capacity (Yang et al., 2024). In animal models, zinc supplementation reduced arsenic and lead toxicity by restoring antioxidant activity and increasing metallothionein expression (Ganger et al., 2016; Prastiya et al., 2023). Zinc supplementation also increased the activities of antioxidant enzymes including superoxide dismutase, glutathione peroxidase, glutathione reductase, and catalase (Kumar et al., 2010), and restored function of other zinc-dependent proteins that were disrupted with arsenic exposure (Cooper et al., 2013; Banerjee et al., 2022; Bastick et al., 2022). Zinc supplementation also prevented cadmium and lead-mediated oxidative damage to the brain (Prasanthi et al., 2010; Brzoska et al., 2021). Among animal models of DNA damage, zinc supplementation reduced cadmium-induced DNA damage in zebrafish (Devarapogu and Asupatri, 2023), and reduced arsenite-enhanced DNA damage in response to ultraviolet radiation exposure in mice (Cooper et al., 2013).

## Associations of zinc status, toxic metals exposure, and health in human studies

There are several lines of evidence in human population studies indicating zinc status and dietary zinc intake are inversely associated with metal toxicity (Talpur et al., 2018). For cadmium exposures, zinc intake is associated with lower cadmium burden in U.S. adults (Vance and Chun, 2015). In this study, increased levels of dietary and serum zinc were associated with a decrease in blood cadmium and an increase in urinary cadmium, suggesting zinc status influenced the absorption, accumulation, and excretion of cadmium. In other studies, dietary zinc intake/status modulated mortality risks associated with cadmium exposure (Kim et al., 2019), cadmium-induced risk of prostate cancer (Bede-Ojimadu et al., 2023), and renal damage (Chen et al., 2018). In children with autistic disorders, over 30% of the study population had zinc deficiency that correlated with high toxic metal burden including lead, cadmium, and arsenic (Yasuda and Tsutsui, 2022). High zinc levels in peripheral blood was associated with protection of workers against occupational exposure to lead, where zinc status inversely correlated with lead concentrations, DNA damage, oxidative stress and lead-induced blood cell membrane aberrations (Wani et al., 2017; Wani et al., 2019).

While human population studies demonstrated the association between zinc status and metal toxicities, to date there is only one intervention study that directly assesses individual toxic metal exposures and responses to zinc supplementation in individuals with chronic toxic metal exposures (NCT03908736, ClinicalTrials.gov). While some zinc supplementation intervention studies have been conducted in regions with populations at risk for toxic metal exposures, the relationship between zinc status and toxic metals exposure has not been examined. More intervention trials are needed to examine the efficacy of zinc supplementation in mitigating metal toxicity in at risk communities. Another ongoing challenge in human studies is the lack of reliable, specific and sensitive biomarkers to accurately identify and evaluate zinc status, particularly in individuals at risk for marginal zinc deficiency (Lowe et al., 2009).

The overall health consequence of toxic metal exposures can include dysfunction to multiple organ systems, resulting in persistent infections (Zheng et al., 2023; Zhang et al., 2024), increased risk for chronic and metabolic diseases (Planchart et al., 2018; Javaid et al., 2021; Pan et al., 2024), neurological disorders (Pamphlett and Bishop, 2023), and cancers (Khoshakhlagh et al., 2024). It is notable that zinc deficiency impacts similar organ systems and is associated with many of the same chronic diseases as toxic metals exposure. Many zinc supplementation studies have demonstrated the protective effects of zinc in similar diseases that are affected by metals toxicity (Li J. et al., 2022). In humans, zinc supplementation lowered the incidence, duration, symptoms, mortality and recovery times in infectious diseases encompassing viral, bacterial, and parasitic pathogens (Maywald and Rink, 2022; Ben Abdallah et al., 2023). In pre-diabetic and diabetic individuals, zinc supplementation improved glycemic control, insulin sensitivity, and reduced inflammatory biomarkers (Wang et al., 2019) and improved risk factors for cardiovascular diseases (Pompano and Boy, 2021). Other clinical studies showed zinc supplementation decreased clinical depression (da Silva et al., 2021; Yosaee et al., 2022), improved neurologic recovery in patients with traumatic brain injury (Young et al., 1996), and improved cognitive function in school children and overweight or obese women (de Moura et al., 2013; de Vargas et al., 2023). While these studies do not address toxic metals exposure, nevertheless accumulating evidence indicates improving zinc status via increased dietary intake or supplementation should confer protection to susceptible individuals, including populations at risk for toxic metal exposures.

## Overall impact of zinc status on health

Work from Dr. Ames and others collectively establish zinc as one of the micronutrients essential for health, in particular via its role in cellular oxidant defense and maintaining DNA integrity. The consequence of zinc inadequacies has a significant impact on human health. Together this work highlights the importance of maintaining adequate micronutrient levels, like zinc in the body to preserve key biological functions and improve many aspects of human health. Large bodies of work from the past several decades underscores the idea originally put forth by Dr. Ames that adequate nutrition, including zinc intake, is essential for optimal health and promoting healthy aging.

## References

[B1] Aimo L. Oteiza P. I. (2006). Zinc deficiency increases the susceptibility of human neuroblastoma cells to lead-induced activator protein-1 activation. Toxicol. Sci. 91 (1), 184–191. 10.1093/toxsci/kfj137 16484283

[B2] Ames B. N. (1999). Micronutrient deficiencies. A major cause of DNA damage. Ann. N. Y. Acad. Sci. 889, 87–106. 10.1111/j.1749-6632.1999.tb08727.x 10668486

[B3] Ames B. N. (2001). DNA damage from micronutrient deficiencies is likely to be a major cause of cancer. Mutat. Res. 475 (1-2), 7–20. 10.1016/s0027-5107(01)00070-7 11295149

[B4] Ames B. N. (2006). Low micronutrient intake May accelerate the degenerative diseases of aging through allocation of scarce micronutrients by triage. Proc. Natl. Acad. Sci. U. S. A. 103 (47), 17589–17594. 10.1073/pnas.0608757103 17101959 PMC1693790

[B5] Ames B. N. (2022). Musings in the twilight of my career. Free Radic. Biol. Med. 178, 219–225. 10.1016/j.freeradbiomed.2021.11.038 34863877

[B6] Ames B. N. Atamna H. Killilea D. W. (2005). Mineral and vitamin deficiencies can accelerate the mitochondrial decay of aging. Mol. Asp. Med. 26 (4-5), 363–378. 10.1016/j.mam.2005.07.007 16102804

[B7] Balali-Mood M. Naseri K. Tahergorabi Z. Khazdair M. R. Sadeghi M. (2021). Toxic mechanisms of five heavy metals: Mercury, lead, chromium, cadmium, and arsenic. Front. Pharmacol. 12, 643972. 10.3389/fphar.2021.643972 33927623 PMC8078867

[B8] Banerjee M. Ferragut Cardoso A. P. Lykoudi A. Wilkey D. W. Pan J. Watson W. H. (2020). Arsenite exposure displaces zinc from ZRANB2 leading to altered splicing. Chem. Res. Toxicol. 33 (6), 1403–1417. 10.1021/acs.chemrestox.9b00515 32274925 PMC7405655

[B9] Banerjee M. Yaddanapudi K. States J. C. (2022). Zinc supplementation prevents mitotic accumulation in human keratinocyte cell lines upon environmentally relevant arsenic exposure. Toxicol. Appl. Pharmacol. 454, 116255. 10.1016/j.taap.2022.116255 36162444 PMC9683715

[B10] Bastick J. C. Banerjee M. States J. C. (2022). Zinc supplementation prevents arsenic-induced dysregulation of ZRANB2 splice function. Environ. Toxicol. Pharmacol. 94, 103921. 10.1016/j.etap.2022.103921 35764259 PMC9945473

[B11] Beaver L. M. Truong L. Barton C. L. Chase T. T. Gonnerman G. D. Wong C. P. (2017). Combinatorial effects of zinc deficiency and arsenic exposure on zebrafish (*Danio rerio*) development. PLoS One 12 (8), e0183831. 10.1371/journal.pone.0183831 28837703 PMC5570330

[B12] Bede-Ojimadu O. Nnamah N. Onuegbu J. Grant-Weaver I. Barraza F. Orakwe J. (2023). Cadmium exposure and the risk of prostate cancer among Nigerian men: effect modification by zinc status. J. Trace Elem. Med. Biol. 78, 127168. 10.1016/j.jtemb.2023.127168 37043921

[B13] Ben Abdallah S. Mhalla Y. Trabelsi I. Sekma A. Youssef R. Bel Haj Ali K. (2023). Twice-daily oral zinc in the treatment of patients with coronavirus disease 2019: a randomized double-blind controlled trial. Clin. Infect. Dis. 76 (2), 185–191. 10.1093/cid/ciac807 36367144

[B14] Bruno R. S. Song Y. Leonard S. W. Mustacich D. J. Taylor A. W. Traber M. G. (2007). Dietary zinc restriction in rats alters antioxidant status and increases plasma F2 isoprostanes. J. Nutr. Biochem. 18 (8), 509–518. 10.1016/j.jnutbio.2006.09.001 17142032

[B15] Brzoska M. M. Kozlowska M. Rogalska J. Galazyn-Sidorczuk M. Roszczenko A. Smereczanski N. M. (2021). Enhanced zinc intake protects against oxidative stress and its consequences in the brain: a study in an *in vivo* rat model of cadmium exposure. Nutrients 13 (2), 478. 10.3390/nu13020478 33572579 PMC7911633

[B16] Bushnell P. J. Levin E. D. (1983). Effects of zinc deficiency on lead toxicity in rats. Neurobehav. Toxicol. Teratol. 5 (3), 283–288. 6877467

[B17] Butt M. S. Iahtisham Ul H. Javed K. Tariq U. (2023). Co-supplementation of zinc and calcium suppresses bio-absorption of lead in sprague dawley rats. Biol. Trace Elem. Res. 201 (3), 1317–1326. 10.1007/s12011-022-03233-3 35399139

[B18] Cao A. L. Beaver L. M. Wong C. P. Hudson L. G. Ho E. (2019). Zinc deficiency alters the susceptibility of pancreatic beta cells (INS-1) to arsenic exposure. Biometals 32 (6), 845–859. 10.1007/s10534-019-00217-0 31542844 PMC6891200

[B19] Chen B. Yu P. Chan W. N. Xie F. Zhang Y. Liang L. (2024). Cellular zinc metabolism and zinc signaling: from biological functions to diseases and therapeutic targets. Signal Transduct. Target Ther. 9 (1), 6. 10.1038/s41392-023-01679-y 38169461 PMC10761908

[B20] Chen X. Wang Z. Zhu G. Nordberg G. F. Ding X. Jin T. (2018). The association between renal tubular dysfunction and zinc level in a Chinese population environmentally exposed to cadmium. Biol. Trace Elem. Res. 186 (1), 114–121. 10.1007/s12011-018-1304-3 29574673

[B21] Cooper K. L. King B. S. Sandoval M. M. Liu K. J. Hudson L. G. (2013). Reduction of arsenite-enhanced ultraviolet radiation-induced DNA damage by supplemental zinc. Toxicol. Appl. Pharmacol. 269 (2), 81–88. 10.1016/j.taap.2013.03.008 23523584 PMC3699402

[B22] Dashner-Titus E. J. Schilz J. R. Alvarez S. A. Wong C. P. Simmons K. Ho E. (2023). Zinc supplementation alters tissue distribution of arsenic in *Mus musculus* . Toxicol. Appl. Pharmacol. 478, 116709. 10.1016/j.taap.2023.116709 37797845 PMC10729601

[B23] da Silva L. E. M. de Santana M. L. P. Costa P. R. F. Pereira E. M. Nepomuceno C. M. M. Queiroz V. A. O. (2021). Zinc supplementation combined with antidepressant drugs for treatment of patients with depression: a systematic review and meta-analysis. Nutr. Rev. 79 (1), 1–12. 10.1093/nutrit/nuaa039 32885249

[B24] de Moura J. E. de Moura E. N. Alves C. X. Vale S. H. Dantas M. M. Silva Ade A. (2013). Oral zinc supplementation May improve cognitive function in schoolchildren. Biol. Trace Elem. Res. 155 (1), 23–28. 10.1007/s12011-013-9766-9 23892699

[B25] Devarapogu R. Asupatri U. R. (2023). Effects of zinc supplementation in mitigating the harmful effects of chronic cadmium exposure in a zebrafish model. Environ. Toxicol. Pharmacol. 100, 104158. 10.1016/j.etap.2023.104158 37236493

[B26] de Vargas L. D. S. Jantsch J. Fontoura J. R. Dorneles G. P. Peres A. Guedes R. P. (2023). Effects of zinc supplementation on inflammatory and cognitive parameters in middle-aged women with overweight or obesity. Nutrients 15 (20), 4396. 10.3390/nu15204396 37892471 PMC10609714

[B27] Ferdigg A. Hopp A. K. Wolf G. Superti-Furga G. (2025). Membrane transporters modulating the toxicity of arsenic, cadmium, and Mercury in human cells. Life Sci. Alliance 8 (2), e202402866. 10.26508/lsa.202402866 39578074 PMC11584324

[B28] Ganger R. Garla R. Mohanty B. P. Bansal M. P. Garg M. L. (2016). Protective effects of zinc against acute arsenic toxicity by regulating antioxidant defense system and cumulative metallothionein expression. Biol. Trace Elem. Res. 169 (2), 218–229. 10.1007/s12011-015-0400-x 26113309

[B29] Gaulke C. A. Rolshoven J. Wong C. P. Hudson L. G. Ho E. Sharpton T. J. (2018). Marginal zinc deficiency and environmentally relevant concentrations of arsenic elicit combined effects on the gut microbiome. mSphere 3 (6), e00521-18. 10.1128/mSphere.00521-18 30518676 PMC6282007

[B30] Guo X. Su W. Li N. Song Q. Wang H. Liang Q. (2022). Association of urinary or blood heavy metals and mortality from all causes, cardiovascular disease, and cancer in the general population: a systematic review and meta-analysis of cohort studies. Environ. Sci. Pollut. Res. Int. 29 (45), 67483–67503. 10.1007/s11356-022-22353-w 35917074

[B31] Hara T. Takeda T. A. Takagishi T. Fukue K. Kambe T. Fukada T. (2017). Physiological roles of zinc transporters: molecular and genetic importance in zinc homeostasis. J. Physiol. Sci. 67 (2), 283–301. 10.1007/s12576-017-0521-4 28130681 PMC10717645

[B32] Hietanen E. Aitio A. Koivusaari U. Kilpio J. Nevalainen T. Narhi M. (1982). Tissue concentrations and interaction of zinc with lead toxicity in rabbits. Toxicology 25 (2-3), 113–127. 10.1016/0300-483x(82)90023-3 7157394

[B33] Ho E. Ames B. N. (2002). Low intracellular zinc induces oxidative DNA damage, disrupts p53, NFkappa B, and AP1 DNA binding, and affects DNA repair in a rat glioma cell line. Proc. Natl. Acad. Sci. U. S. A. 99 (26), 16770–16775. 10.1073/pnas.222679399 12481036 PMC139219

[B34] Ho E. Courtemanche C. Ames B. N. (2003). Zinc deficiency induces oxidative DNA damage and increases p53 expression in human lung fibroblasts. J. Nutr. 133 (8), 2543–2548. 10.1093/jn/133.8.2543 12888634

[B36] Hudson L. G. Dashner-Titus E. J. MacKenzie D. (2025). Zinc as a mechanism-based strategy for mitigation of metals toxicity. Curr. Environ. Health Rep. 12 (1), 5. 10.1007/s40572-025-00474-x 39827326 PMC11742765

[B37] Jamieson J. A. Taylor C. G. Weiler H. A. (2006). Marginal zinc deficiency exacerbates bone lead accumulation and high dietary zinc attenuates lead accumulation at the expense of bone density in growing rats. Toxicol. Sci. 92 (1), 286–294. 10.1093/toxsci/kfj201 16624848

[B38] Javaid A. Akbar I. Javed H. Khan U. Iftikhar H. Zahra D. (2021). Role of heavy metals in diabetes: mechanisms and treatment strategies. Crit. Rev. Eukaryot. Gene Expr. 31 (3), 65–80. 10.1615/CritRevEukaryotGeneExpr.2021037971 34369715

[B39] Joray M. L. Yu T. W. Ho E. Clarke S. L. Stanga Z. Gebreegziabher T. (2015). Zinc supplementation reduced DNA breaks in Ethiopian women. Nutr. Res. 35 (1), 49–55. 10.1016/j.nutres.2014.10.006 25491347 PMC4466114

[B40] Khoshakhlagh A. H. Mohammadzadeh M. Gruszecka-Kosowska A. (2024). The preventive and carcinogenic effect of metals on cancer: a systematic review. BMC Public Health 24 (1), 2079. 10.1186/s12889-024-19585-5 39090615 PMC11293075

[B41] Kim K. Melough M. M. Sakaki J. R. Ha K. Marmash D. Noh H. (2019). Association between urinary cadmium to zinc intake ratio with adult mortality in a Follow-Up study of NHANES 1988-1994 and 1999-2004. Nutrients 12 (1), 56. 10.3390/nu12010056 31878194 PMC7019386

[B42] Koyama H. Kamogashira T. Yamasoba T. (2024). Heavy metal exposure: molecular pathways, clinical implications, and protective strategies. Antioxidants (Basel) 13 (1), 76. 10.3390/antiox13010076 38247500 PMC10812460

[B43] Kumar A. Malhotra A. Nair P. Garg M. Dhawan D. K. (2010). Protective role of zinc in ameliorating arsenic-induced oxidative stress and histological changes in rat liver. J. Environ. Pathol. Toxicol. Oncol. 29 (2), 91–100. 10.1615/jenvironpatholtoxicoloncol.v29.i2.30 20932244

[B44] Lee S. R. (2018). Critical role of zinc as either an antioxidant or a prooxidant in cellular systems. Oxid. Med. Cell Longev. 2018, 9156285. 10.1155/2018/9156285 29743987 PMC5884210

[B45] Li J. Cao D. Huang Y. Chen B. Chen Z. Wang R. (2022). Zinc intakes and health outcomes: an umbrella review. Front. Nutr. 9, 798078. 10.3389/fnut.2022.798078 35211497 PMC8861317

[B46] Li Z. Liu Y. Wei R. Yong V. W. Xue M. (2022). The important role of zinc in neurological diseases. Biomolecules 13 (1), 28. 10.3390/biom13010028 36671413 PMC9855948

[B47] Lowe N. M. Fekete K. Decsi T. (2009). Methods of assessment of zinc status in humans: a systematic review. Am. J. Clin. Nutr. 89 (6), 2040S–2051S. 10.3945/ajcn.2009.27230G 19420098

[B48] Lowe N. M. Hall A. G. Broadley M. R. Foley J. Boy E. Bhutta Z. A. (2024). Preventing and controlling zinc deficiency across the life course: a call to action. Adv. Nutr. 15 (3), 100181. 10.1016/j.advnut.2024.100181 38280724 PMC10882121

[B49] Maywald M. Rink L. (2022). Zinc in human health and infectious diseases. Biomolecules 12 (12), 1748. 10.3390/biom12121748 36551176 PMC9775844

[B50] NIH-ODS (2022). Zinc - Fact sheet for health professionals. Bethesda, MD: National Institute of Health Office of Dietary Supplements. Available online at: https://ods.od.nih.gov/factsheets/Zinc-HealthProfessional/ (Accessed April 21, 2025).

[B51] Ocampo D. Damon L. J. Sanford L. Holtzen S. E. Jones T. Allen M. A. (2024). Cellular zinc status alters chromatin accessibility and binding of p53 to DNA. Life Sci. Alliance 7 (9), e202402638. 10.26508/lsa.202402638 38969365 PMC11231577

[B52] Oteiza P. I. Olin K. L. Fraga C. G. Keen C. L. (1995). Zinc deficiency causes oxidative damage to proteins, lipids and DNA in rat testes. J. Nutr. 125 (4), 823–829. 10.1093/jn/125.4.823 7722683

[B53] Ozoani H. Ezejiofor A. N. Okolo K. O. Orish C. N. Cirovic A. Cirovic A. (2024). Ameliorative effects of Zn and Se supplementation on heavy metal mixture burden *via* increased renal metal excretion and restoration of redoxo-inflammatory alterations. Biol. Trace Elem. Res. 202 (2), 643–658. 10.1007/s12011-023-03709-w 37231320

[B54] Pabis K. Gundacker C. Giacconi R. Basso A. Costarelli L. Piacenza F. (2018). Zinc supplementation can reduce accumulation of cadmium in aged metallothionein transgenic mice. Chemosphere 211, 855–860. 10.1016/j.chemosphere.2018.08.017 30103140

[B55] Pamphlett R. Bishop D. P. (2023). The toxic metal hypothesis for neurological disorders. Front. Neurol. 14, 1173779. 10.3389/fneur.2023.1173779 37426441 PMC10328356

[B56] Pan Z. Gong T. Liang P. (2024). Heavy metal exposure and cardiovascular disease. Circ. Res. 134 (9), 1160–1178. 10.1161/CIRCRESAHA.123.323617 38662861

[B57] Passarelli S. Free C. M. Shepon A. Beal T. Batis C. Golden C. D. (2024). Global estimation of dietary micronutrient inadequacies: a modelling analysis. Lancet Glob. Health 12 (10), e1590–e1599. 10.1016/S2214-109X(24)00276-6 39218000 PMC11426101

[B58] Planchart A. Green A. Hoyo C. Mattingly C. J. (2018). Heavy metal exposure and metabolic syndrome: evidence from human and model system studies. Curr. Environ. Health Rep. 5 (1), 110–124. 10.1007/s40572-018-0182-3 29460222 PMC6053628

[B59] Pompano L. M. Boy E. (2021). Effects of dose and duration of zinc interventions on risk factors for type 2 diabetes and cardiovascular disease: a systematic review and meta-analysis. Adv. Nutr. 12 (1), 141–160. 10.1093/advances/nmaa087 32722790 PMC7850144

[B60] Prasanthi R. P. Devi C. B. Basha D. C. Reddy N. S. Reddy G. R. (2010). Calcium and zinc supplementation protects lead (Pb)-induced perturbations in antioxidant enzymes and lipid peroxidation in developing mouse brain. Int. J. Dev. Neurosci. 28 (2), 161–167. 10.1016/j.ijdevneu.2009.12.002 20036325

[B61] Prastiya R. A. Sardjito T. Nabila T. R. N. Azizah H. I. N. Saputro A. L. Sasi S. M. (2023). Zinc and alpha-tocopherol protect the antral follicles and endogenous antioxidants of female albino rats (*Rattus norvegicus*) against lead toxicity. J. Trace Elem. Med. Biol. 80, 127284. 10.1016/j.jtemb.2023.127284 37657266

[B62] Reider C. A. Chung R. Y. Devarshi P. P. Grant R. W. Hazels Mitmesser S. (2020). Inadequacy of immune health nutrients: intakes in US adults, the 2005-2016 NHANES. Nutrients 12 (6), 1735. 10.3390/nu12061735 32531972 PMC7352522

[B63] Sharif R. Thomas P. Zalewski P. Fenech M. (2012). Zinc deficiency or excess within the physiological range increases genome instability and cytotoxicity, respectively, in human oral keratinocyte cells. Genes Nutr. 7 (2), 139–154. 10.1007/s12263-011-0248-4 21935692 PMC3316759

[B64] Sharma P. Wong C. P. Ho E. Sampath H. (2024). Catalytic activity of OGG1 is impaired by zinc deficiency. DNA Repair (Amst) 134, 103628. 10.1016/j.dnarep.2024.103628 38228016 PMC10851324

[B65] Shim Y. K. Lewin M. D. Ruiz P. Eichner J. E. Mumtaz M. M. (2017). Prevalence and associated demographic characteristics of exposure to multiple metals and their species in human populations: the United States NHANES, 2007-2012. J. Toxicol. Environ. Health A 80 (9), 502–512. 10.1080/15287394.2017.1330581 28703686 PMC5693367

[B66] Song Y. Chung C. S. Bruno R. S. Traber M. G. Brown K. H. King J. C. (2009a). Dietary zinc restriction and repletion affects DNA integrity in healthy men. Am. J. Clin. Nutr. 90 (2), 321–328. 10.3945/ajcn.2008.27300 19515738 PMC2709309

[B67] Song Y. Elias V. Loban A. Scrimgeour A. G. Ho E. (2010a). Marginal zinc deficiency increases oxidative DNA damage in the prostate after chronic exercise. Free Radic. Biol. Med. 48 (1), 82–88. 10.1016/j.freeradbiomed.2009.10.030 19836448 PMC4090116

[B68] Song Y. Elias V. Wong C. P. Scrimgeour A. G. Ho E. (2010b). Zinc transporter expression profiles in the rat prostate following alterations in dietary zinc. Biometals 23 (1), 51–58. 10.1007/s10534-009-9266-8 19760107 PMC4152227

[B69] Song Y. Leonard S. W. Traber M. G. Ho E. (2009b). Zinc deficiency affects DNA damage, oxidative stress, antioxidant defenses, and DNA repair in rats. J. Nutr. 139 (9), 1626–1631. 10.3945/jn.109.106369 19625698 PMC3151020

[B70] Sugimoto R. Lee L. Tanaka Y. Morita Y. Hijioka M. Hisano T. (2024). Zinc deficiency as a general feature of cancer: a review of the literature. Biol. Trace Elem. Res. 202 (5), 1937–1947. 10.1007/s12011-023-03818-6 37658952 PMC10955002

[B71] Talpur S. Afridi H. I. Kazi T. G. Talpur F. N. (2018). Interaction of lead with calcium, iron, and zinc in the biological samples of malnourished children. Biol. Trace Elem. Res. 183 (2), 209–217. 10.1007/s12011-017-1141-9 28861860

[B72] Tamura Y. (2021). The role of zinc homeostasis in the prevention of diabetes mellitus and cardiovascular diseases. J. Atheroscler. Thromb. 28 (11), 1109–1122. 10.5551/jat.RV17057 34148917 PMC8592709

[B73] Tchounwou P. B. Yedjou C. G. Patlolla A. K. Sutton D. J. (2012). Heavy metal toxicity and the environment. Exp. Suppl. 101, 133–164. 10.1007/978-3-7643-8340-4_6 22945569 PMC4144270

[B74] Trojan H. E. Rink L. Jakobs J. (2024). Zinc deficiency exacerbates lead-induced Interleukin-2 suppression by regulating CREM expression. Int. J. Mol. Sci. 26 (1), 254. 10.3390/ijms26010254 39796118 PMC11720117

[B75] Ugwuja E. I. Vincent N. Ohayi S. R. (2020). Zinc ameliorates lead toxicity by reducing body Pb burden and restoring Pb-induced haematological and biochemical derangements. Toxicol. Res. Appl. 4, 10.1177/2397847320956562

[B76] Vance T. M. Chun O. K. (2015). Zinc intake is associated with lower cadmium burden in U.S. adults. J. Nutr. 145 (12), 2741–2748. 10.3945/jn.115.223099 26491124

[B77] Waalkes M. P. (1986). Effect of dietary zinc deficiency on the accumulation of cadmium and metallothionein in selected tissues of the rat. J. Toxicol. Environ. Health 18 (2), 301–313. 10.1080/15287398609530870 3712492

[B78] Waalkes M. P. Kovatch R. Rehm S. (1991). Effect of chronic dietary zinc deficiency on cadmium toxicity and carcinogenesis in the Male wistar [hsd: (WI)BR] rat. Toxicol. Appl. Pharmacol. 108 (3), 448–456. 10.1016/0041-008x(91)90091-r 2020969

[B79] Wang X. Wu W. Zheng W. Fang X. Chen L. Rink L. (2019). Zinc supplementation improves glycemic control for diabetes prevention and management: a systematic review and meta-analysis of randomized controlled trials. Am. J. Clin. Nutr. 110 (1), 76–90. 10.1093/ajcn/nqz041 31161192

[B80] Wani A. L. Ahmad A. Shadab G. G. Usmani J. A. (2017). Possible role of zinc in diminishing lead-related occupational stress-a zinc nutrition concern. Environ. Sci. Pollut. Res. Int. 24 (9), 8682–8691. 10.1007/s11356-017-8569-5 28204951

[B81] Wani A. L. Ansari M. O. Ahmad M. F. Parveen N. Siddique H. R. Shadab G. (2019). Influence of zinc levels on the toxic manifestations of lead exposure among the occupationally exposed workers. Environ. Sci. Pollut. Res. Int. 26 (32), 33541–33554. 10.1007/s11356-019-06443-w 31583521

[B82] Wani A. L. Hammad Ahmad Shadab G. G. Afzal M. (2021). Lead and zinc interactions - an influence of zinc over lead related toxic manifestations. J. Trace Elem. Med. Biol. 64, 126702. 10.1016/j.jtemb.2020.126702 33285442

[B83] WHO (2020). 10 chemicals of public health concern. Available online at: https://www.who.int/news-room/photo-story/detail/10-chemicals-of-public-health-concern (Accessed April 21, 2025).

[B84] Wong C. P. Dashner-Titus E. J. Alvarez S. C. Chase T. T. Hudson L. G. Ho E. (2019). Zinc deficiency and arsenic exposure can act both independently or cooperatively to affect zinc status, oxidative stress, and inflammatory response. Biol. Trace Elem. Res. 191 (2), 370–381. 10.1007/s12011-019-1631-z 30635848 PMC6625954

[B85] Wong C. P. Magnusson K. R. Sharpton T. J. Ho E. (2021). Effects of zinc status on age-related T cell dysfunction and chronic inflammation. Biometals 34 (2), 291–301. 10.1007/s10534-020-00279-5 33392795 PMC8559905

[B86] Xu Y. Li A. Li X. Deng X. Gao X. J. (2023). Zinc deficiency induces inflammation and apoptosis *via* oxidative stress in the kidneys of mice. Biol. Trace Elem. Res. 201 (2), 739–750. 10.1007/s12011-022-03166-x 35211842

[B87] Yan M. Song Y. Wong C. P. Hardin K. Ho E. (2008). Zinc deficiency alters DNA damage response genes in normal human prostate epithelial cells. J. Nutr. 138 (4), 667–673. 10.1093/jn/138.4.667 18356318 PMC4152237

[B88] Yang R. Roshani D. Gao B. Li P. Shang N. (2024). Metallothionein: a comprehensive review of its classification, structure, biological functions, and applications. Antioxidants (Basel) 13 (7), 825. 10.3390/antiox13070825 39061894 PMC11273490

[B89] Yasuda H. Tsutsui T. (2022). Metallomics analysis for early assessment and individualized intervention of neurodevelopmental disorders. Metallomics 14 (9), mfac067. 10.1093/mtomcs/mfac067 36087072

[B90] Yosaee S. Clark C. C. T. Keshtkaran Z. Ashourpour M. Keshani P. Soltani S. (2022). Zinc in depression: from development to treatment: a comparative/dose response meta-analysis of observational studies and randomized controlled trials. Gen. Hosp. Psychiatry 74, 110–117. 10.1016/j.genhosppsych.2020.08.001 32829928

[B91] Young B. Ott L. Kasarskis E. Rapp R. Moles K. Dempsey R. J. (1996). Zinc supplementation is associated with improved neurologic recovery rate and visceral protein levels of patients with severe closed head injury. J. Neurotrauma 13 (1), 25–34. 10.1089/neu.1996.13.25 8714860

[B92] Yu H. T. Zhen J. Leng J. Y. Cai L. Ji H. L. Keller B. B. (2021). Zinc as a countermeasure for cadmium toxicity. Acta Pharmacol. Sin. 42 (3), 340–346. 10.1038/s41401-020-0396-4 32284539 PMC8027184

[B93] Zhang H. Wang J. Zhang K. Shi J. Gao Y. Zheng J. (2024). Association between heavy metals exposure and persistent infections: the mediating role of immune function. Front. Public Health 12, 1367644. 10.3389/fpubh.2024.1367644 39104887 PMC11298456

[B94] Zhang Q. Xue Y. Fu Y. Bao B. Guo M. Y. (2022). Zinc deficiency aggravates oxidative stress leading to inflammation and fibrosis in lung of mice. Biol. Trace Elem. Res. 200 (9), 4045–4057. 10.1007/s12011-021-03011-7 34739677

[B95] Zheng K. Zeng Z. Tian Q. Huang J. Zhong Q. Huo X. (2023). Epidemiological evidence for the effect of environmental heavy metal exposure on the immune system in children. Sci. Total Environ. 868, 161691. 10.1016/j.scitotenv.2023.161691 36669659

[B96] Zyba S. J. Shenvi S. V. Killilea D. W. Holland T. C. Kim E. Moy A. (2017). A moderate increase in dietary zinc reduces DNA strand breaks in leukocytes and alters plasma proteins without changing plasma zinc concentrations. Am. J. Clin. Nutr. 105 (2), 343–351. 10.3945/ajcn.116.135327 28003206 PMC5267297

